# Linking Variability of Leukocyte Lipid Metabolism to Circulating Lipids, Lipoprotein Composition, and Cardiovascular Risk in the Finnish Adult Population

**DOI:** 10.1161/ATVBAHA.125.322801

**Published:** 2025-06-19

**Authors:** Iryna Hlushchenko, Siina Pamilo, Mohammad Majharul Islam, Iris Aino Karoliina Lähdeniemi, Max Tamlander, Samuli Ripatti, Simon Georg Pfisterer

**Affiliations:** Department of Anatomy, Faculty of Medicine (I.H., S.P., M.M.I., I.A.K.L., S.G.P.), University of Helsinki.; Institute for Molecular Medicine Finland, Helsinki Institute of Life Science (M.T., S.R.), University of Helsinki.; Clinicum, Department of Public Health (S.R.), University of Helsinki.; MONCYTE Health, Helsinki, Finland (S.P., I.A.K.L., S.G.P.).; Broad Institute of the Massachusetts Institute of Technology and Harvard, Cambridge, MA (S.R.).

**Keywords:** cardiovascular diseases, lipid droplets, lipids, lipoproteins, monocytes, myocardial infarction, receptors, LDL

## Abstract

**BACKGROUND:**

Interindividual differences in outcomes of lipid-lowering therapy are well known. Here, we aimed to characterize how alterations in cellular lipid uptake, storage, and utilization pathways may contribute to different treatment outcomes.

**METHODS:**

We performed an observational case-control biobank study quantifying leukocyte LDL (low-density lipoprotein) uptake and lipid storage with an automated multiplexed analysis pipeline for 133 statin recipients and 135 control subjects from the FINRISK 2012 study, a Finnish population survey on risk factors on chronic noncommunicable diseases. Individual cellular readouts as well as their combinations, which we called lipid trafficking scores, were then correlated to blood lipid values and health outcomes of the study participants.

**RESULTS:**

Of individuals receiving high-intensity statin therapy, those with lower lipid trafficking scores displayed higher circulating concentrations of several proatherogenic lipoproteins and had higher odds for myocardial infarction and stroke when compared with the rest of the subjects with equivalent treatment. Most subjects with a poor lipid trafficking score did not reach low-density lipoprotein cholesterol target levels on statin monotherapy. Combining lipid trafficking score with a polygenic risk score for low-density lipoprotein cholesterol strengthened the association with a proatherogenic lipoprotein profile.

**CONCLUSIONS:**

Our results indicate that quantification of cellular lipid trafficking can aid in treatment selection and risk assessment in dyslipidemia.

**Highlights**

- We present an analysis platform providing systematic insight into cellular processes underlying dyslipidemia and cardiovascular disease from human leukocytes.
- Alterations in monocyte LDL (low-density lipoprotein) uptake and lipid storage are associated with different treatment outcomes for statin recipients.
- Recipients of high-intensity statin medication with low monocyte LDL uptake and lipid handling capacity display elevations in proatherogenic lipoproteins and increased cardiovascular disease risk.
- Quantification of cellular processes from human leukocytes can aid in treatment selection and assessment of residual cardiovascular disease risk.

Hypercholesterolemia is a global health problem and the leading cause of cardiovascular disease (CVD).^1^ Although multiple treatment options exist, such as statins, ezetimibe, or PCSK9 (proprotein convertase subtilisin/kexin type 9) inhibitors (PCSK9i), a significant fraction of high-risk patients does not achieve desired LDL (low-density lipoprotein) cholesterol (LDL-C) levels.^2–8^ The reasons for poor goal attainment are multifactorial, including low adherence to the prescribed therapy, as well as challenges imposed through healthcare systems and reimbursement rules.^9–12^ Interindividual variation in treatment outcomes has been reported in several studies,^3,13,14^ including those in which adherence has been monitored closely.^15^ Therefore, it appears possible that biological variation in cellular pathways underlying drug action might represent an important contributing factor to goal attainment, but lacks experimental data to receive recognition.

Statins and PCSK9i influence cellular lipid trafficking (LT) via different mechanisms. Although PCSK9i block degradation of the LDLR (low-density lipoprotein receptor), statins evoke cellular cholesterol depletion and increased LDLR expression.^16–18^ In both cases, more abundant plasma membrane LDLR results in increased LDL uptake and clearance from the blood. Cell surface abundance of LDLR is linked to cellular lipid homeostasis, with excess cholesterol and lipid storage resulting in reduced LDLR expression.^19,20^ Increased lipid droplet formation has been observed in individuals with familial hypercholesterolemia (FH),^21,22^ and in mouse models circulating lipid-laden monocytes have been shown to infiltrate early atherosclerotic lesions and contribute to nascent atherosclerosis development.^23^ Moreover, studies have linked hyperlipidemia and lipid droplet formation in human monocytes.^21,24^ Consequently, quantification of cellular lipid droplets may provide important insight into the biological variation of these cellular processes which have been related to dyslipidemia and CVD previously.

For patients with FH, alterations in cellular LDL uptake have been linked to treatment outcomes for statin and PCSK9i medication.^25–29^ Initially, cellular LDL uptake studies were designed to identify patients with FH.^30^ However, differentiation of patients with FH from control individuals was difficult, due to interindividual variability in both groups.^31^

Previously, we have established a multiplexed automated analysis pipeline for systematic and detailed quantification of cellular LDL uptake and lipid storage in leukocyte subpopulations under different cholesterol depletion states. It provided 8 readouts on LDL uptake, 12 readouts on lipid storage, and 6 derived measurements on lipid mobilization for each subject, with lipid mobilization reflecting lipid droplet depletion on lipid starvation.^25^ We demonstrated that monocytes display more pronounced responses in both LDL uptake and lipid storage than lymphocytes and that individuals with identical LDLR mutations display highly divergent LDL uptake profiles which are associated with achieved LDL-C on statin therapy.^25^ This highlights that variations in cellular LT pathways are likely widespread, however, it is yet unclear whether they are relevant for lipid-lowering treatment outcomes and CVD progression in the general population. Human leukocyte and liver LDLR expression are highly correlated,^32^ indicating that leukocyte-derived cellular readouts may be utilized as surrogate readouts for hepatocytes to assess biological variation in the context of dyslipidemia. This is supported by previous studies showing a correlation between leukocyte LDL uptake and circulating LDL-C for patients with FH.^25,27–29^

The main objective of this study was to elucidate how LDL uptake and lipid storage in leukocyte subpopulations associate with circulating LDL-C and different lipoprotein subclasses in statin users from the general population, versus subjects not on lipid-lowering therapy. Secondary goals were to examine if poor LT profiles are associated with increased cardiovascular risk and to investigate the interrelationship of cellular and genetic risk scores.

## Methods

This study was conducted in accordance with the Strengthening the Reporting of Observational Studies in Epidemiology guidelines.^33^ The Strengthening the Reporting of Observational Studies in Epidemiology checklist was utilized to ensure comprehensive and transparent reporting of the study’s design, conduct, and findings (Supplemental Material: Strengthening the Reporting of Observational Studies in Epidemiology checklist).

### Data Availability

The data used in this research includes sensitive personal health information and is, therefore, under restricted access. Biological samples and linked laboratory lipid values, questionnaire answers, and biometrics as well as genetic data underlying this article were provided by The National Institute for Health and Welfare (THL) Biobank (www.thl.fi/biobank) and used under Biobank agreement THLBB2020_7. Access to the data can be obtained from the THL Biobank through the standard application procedure. The register-based data including diagnoses, causes of death, and medication purchase history used in this study can be obtained from the Finnish Social and Health Data Permit Authority Findata by submitting a data permit application. Description of the register data used in the study (names of registers and attribute codes) can be obtained from the corresponding author.

### Study Design and Group Definitions

In a previous study, we observed a strong negative correlation between LDL uptake and circulating LDL-C in patients with heterozygous FH on statin monotherapy.^25^ Here, we aimed to determine whether the observed correlation would also be present in statin recipients without the heterozygous FH mutation. We estimated that a sample size of 200 would yield a statistical power of ≥80% for detecting even weak correlations (*r*≥0.2) in statin recipients.^34^ Therefore, we selected from the Biobank database 200 recipients of lipid-lowering therapy and 200 matched controls from the FINRISK 2012 population survey for whom frozen peripheral blood mononuclear cell (PBMC) samples were stored in THL Biobank. Each group was chosen to include subjects with a broad range of LDL-C values and equal sex distribution; medication for blood pressure or diabetes was avoided when possible. To reduce confounding effects we matched the controls to the cases manually based on age, sex, and body mass index (BMI), after filtering out the larger pools for case population and control population. Direct matching to each case was performed. One PBMC cryovial per each of the 400 selected individuals was then retrieved from the THL Biobank.

We obtained laboratory lipid values (total cholesterol, LDL-C, triglycerides, APOB, APO A_1_), age, sex, BMI, waist, and hip circumference for all of the selected individuals. Nuclear magnetic resonance (NMR) metabolomics values were available for 398 subjects and polygenic risk scores could be calculated for 394 participants. For optimal performance of the cellular assays, 1.6 million viable cells were required per each subject. We could obtain only 1 tube of cryopreserved PBMCs stored in the Biobank for each subject. The amount of viable cells per subject ranged from zero to 5.5 million, therefore we could obtain reliable cellular readout results from 275 samples (135 controls and 140 cases).

Our initial case-control selection was based on the questionnaire answer for taking lipid-lowering medication and did not discern between medication type or dose. To gain more insight into the used lipid-lowering medication, we utilized drug purchase history for 2002 to 2019 obtained from the Finnish Social and Health Data Permit Authority Findata for all the subjects. The data included purchase dates, anatomic therapeutic chemical codes for active ingredients, and a Nordic article number (VNR) for specific articles of medicine as well as number of articles bought at each instance. We then selected subjects who purchased lipid-lowering medication during 6 months before PBMC sampling. The last 2 purchases before sampling defined the medication type. This strategy resulted in selection of 202 subjects of whom 191 were on statin monotherapy (simvastatin n=127, atorvastatin n=30, rosuvastatin n=22, fluvastatin n=3, lovastatin n=3, pravastatin n=6), 9 were on combination therapy or took other lipid-lowering medication (ezetimibe=2, fenofibrate=1, statin/ezetimibe=5, statin/ezetimibe/fenofibrate=1), and 2 subjects have switched statin type during the target time period. Because 94% of subjects were on either simvastatin, rosuvastatin, or atorvastatin monotherapy we focused only on these 3 statin types in the further subgroup formation.

We further extracted the purchased statin doses from the same pharmacy records. To see how associations will change with statin potency we formed an additional statin group enriched for high-intensity statins (HIS). The selection was based on previously reported LDL-C reductions from baseline achieved with different doses of simvastatin, rosuvastatin, and atorvastatin.^35–37^ The HIS group contains statins with over 40% LDL-C reduction potential from baseline which can be achieved with rosuvastatin 5 to 40 mg, or atorvastatin 10 to 80 mg, which resulted in a group size of 39 subjects.

Therapy adherence was estimated by dividing the number of tablets in the package bought before blood sampling by the number of days elapsed until the next purchase. The average adherence was as follows (mean±SEM): statin group 0.86±0.03 and HIS group 0.83±0.04. Overall, 55% to 62% of subjects in each analyzed group reported having a history of taking blood pressure medications; additionally, 4% of controls and 23% to 26% of statin recipients reported using diabetes medication (Table S1).

We have conducted the initial association analyses for the subjects for whom cellular data and lipid values were available. Additional subjects needed to be removed in relevant figures because of a lack of NMR metabolomics values (2) and a lack of genetic data for LDL polygenic risk score (LDL-PRS) calculations (3; Figure S1). Please see the Major Resources Table in the Supplemental Material.

### Definition of Adverse Cardiovascular Events

CVD events were defined as myocardial infarction or ischemic stroke specified by I2X and I6X diagnosis codes from the *International Statistical Classification of Diseases and Related Health Problems*, *10th Revision* (Supplemental Methods). Both prevalent and incident CVD events were included and were extracted from hospital admission and primary care electronic health records.

### Human Subject Samples

PBMC samples were cryopreserved in 2012 as a part of Finnish population survey, FINRISK 2012, with written consent and ethical approval of the Hospital District of Helsinki and Uusimaa (permit 162/13/03/00/2011).^38^ The FINRISK 2012 study was performed in accordance with the principles outlined in the Declaration of Helsinki–ethical principles for medical research involving human subjects. The FINRISK 2012 sample collection was then transferred to THL Biobank in 2015. Frozen PBMC samples, pseudonymized donor-linked lipid values, and survey data were obtained then from THL Biobank (www.thl.fi/biobank) and used under Biobank agreement (THLBB2020_7). A mixture of PBMCs from 4 healthy anonymous donors were used as standards for assay measurements. These cells were isolated by density-gradient centrifugation from buffy coat samples obtained through the Finnish Red Cross Blood Service (56/2019).

### PBMC Recovery

Cryopreserved samples were thawed and cell viability was determined using trypan blue exclusion technique. Cells were then seeded into 96-well plates for 24-hour incubation with lipid-rich (R) medium (RPMI-1640 supplemented with 2 mM l-glutamine, 100 units/mL potassium penicillin, 100 µg/mL streptomycin sulfate, 5 mM HEPES and 1 mM sodium pyruvate) containing 10% FBS or lipid-poor (P) medium containing 5% lipoprotein-deprived serum. The cellular assays were performed in 384-well plate format on the next day.

### LDL Uptake and Lipid Droplet Assays

Fluorescent labeling of low-density lipoprotein particles with DiI (1,1'-dioctadecyl-3,3,3',3'-tetramethylindocarbocyanine perchlorate),^39–41^ LDL uptake, and lipid droplet assays^25^ were described previously. DiI-LDL was spiked into the growth media to achieve a final concentration of 30 μg/mL, and the cells were further incubated for 1 hour at 37 °C. Subsequently, the cells were washed and resuspended with serum-free growth medium. Next, using an open-source robotic platform (Opentrons, NY), the cells were transferred and adhered to coated 384-well imaging plates. Afterward, cells were fixed with 4% PFA, washed, and stained with nuclear marker DAPI (4',6-diamidino-2-phenylindole; 5 μg/mL) and cytoplasm marker CellMask DeepRed (0.5 μg/mL). For lipid droplet analysis, the cells were fixed and stained in the same manner as in the LDL uptake assay except that the staining solution contained 5 μg/mL DAPI and 1 μg/mL LD540 (Princeton BioMolecular Research).^42^ Images were acquired for both assays with a PerkinElmer OperaPhenix automated spinning disc confocal microscope using a 40× or 63× water immersion objective. Each assay was repeated twice and 2 PBMC standard samples were included in every experiment. Each analysis batch included matched cases and controls.

### Image Processing and Quantification

Raw confocal 3-dimensional image stacks were automatically deconvolved and collapsed into the maximum intensity projections using a custom-made Python tool (https://github.com/lopaavol/OPutils). Then cell nuclei, cytoplasm, and organelles were segmented automatically using CellProfiler.^43^ Several images from each plate were sampled and inspected by the experimenter for potential segmentation errors to ensure segmentation quality and to filter out imaging artifacts. Cell types were defined based on the measured cell area with cells under 115 µm^2^ classified as lymphocytes and the rest as monocytes. This cutoff value was previously defined based on CD14 and CD3 staining.^25^

### Genotyping, Imputation, and LDL-PRS

We obtained genotypes for 394 of our 400 study individuals from THL Biobank for which single nucleotide polymorphism array and centrally imputed genotype data were available. The FINRISK 2012 samples were genotyped using the Illumina HumanCoreExome-24 v1.1 and Affymetrix Axiom FinnGen1.r1 and FinnGen1.r2 arrays. The genotype imputation workflow with preimputation and postimputation quality control steps was carried out as described in the following protocol: dx.doi.org/10.17504/protocols.io.xbgfijw.

We used PLINK 2.0^44^ (www.cog-genomics.org/plink/2.0/) to first convert PED and MAP files obtained from THL Biobank to BED, BIM, and FAM files and then calculated a genome-wide LDL-PRS using weights for a LDL-PRS calculated in FinnGen^45^ study Data Freeze 7 (https://www.pgscatalog.org/score/PGS002764/). Our final LDL-PRS included 1 086 403 variants and could be calculated to 394 out of 400 subjects. Next, we normalized LDL-PRSs to the 0 to 1 range. Contrary to cellular scores, LDL-PRS was positively associated with LDL-C levels in our study and in previously reported FINIRSK 2012 cohort analysis.^46^ Therefore, to allow for the comparability of LDL-PRS to cellular LT scores and their further integration, we inverted LDL-PRS for all the downstream analyses.

### Subject Health Data Retrieval

General information about subjects’ age, sex, weight, BMI, waist and hip circumference, lifestyle, and medication status (lipid-lowering, diabetes, and blood pressure), as well as standard laboratory lipid values and NMR metabolomics values, were retrieved in conjunction with samples from THL Biobank. In addition, the pseudonymized medical records related to cardiometabolic disorders for each subject were obtained for years 2002 to 2019 from Finnish primary care register (Avohilmo) and national social and healthcare data collection and reporting system (Hilmo). Records of purchase and reimbursement of lipid-lowering medications for years 2002 to 2019 were obtained from the Social Insurance Institution of Finland (Kela). All data extraction procedures were done under permit (THL/4640/14.02.00/2020) and handled by Findata.

### Data Processing and Statistical Analysis

Data were processed and visualized using Python standard libraries (Python Software Foundation, www.python.org) and the following packages: Pandas,^47^ Numpy,^48^ Scipy,^49^ Matplotlib,^50^ Scikit-Learn,^51^ Seaborn.^52^ Single-cell readouts were filtered for cell size and mean intensity outliers with 5×SD from the mean being the cutoff point. On average several hundred monocytes and several thousand lymphocytes were quantified in each well. Wells with <50 cells were discarded from the analysis. We further obtained mean-per-well values for intensity and organelle numbers. For each of the lipid-rich and lipid-poor conditions, we analyzed 4 wells in 2 independent experiments. Absolute values were then normalized to the included control samples. Final readouts were computed by averaging the normalized values from the 8 wells. The lipid mobilization score (LiM) represents the average of the fold change of lipid droplet number, area of lipid droplet–positive cells as well as the percentage of lipid droplet–positive cells in lipid-rich versus poor conditions, derived from 8 wells. LDL uptake and lipid mobilization for the entire data set were additionally rescaled to the 0 to 1 range before combining into LT scores. The LT scores are an average of the LiM with the LDL uptake readouts (LDL-Int and LDL-No) in lipid-rich (LT-R) or poor conditions (LT-P), or LDL uptake readouts in lipid-rich and -poor conditions (LT).

Statistical analyses were performed with python package scipy.stats. Normality tests (Shapiro-Wilk test) were conducted for age, BMI, blood lipid values, our cellular readouts, and the NMR data. Because none of the tested variables were normally distributed, we employed nonparametric tests in our analysis. For pairwise comparisons of group means reported in the Table, the Mann-Whitney *U* test was used. To determine the correlations between cellular scores and lipid values the Spearman rank-order correlation coefficients and corresponding asymptotic *P* values were calculated. To test the difference between lipid species concentrations in controls versus subgroups of statin recipients (Figure 3D) we used Mann-Whitney *U* test. Odds ratios (ORs) were calculated using 2×2 contingency tables and corresponding *P* values were calculated using Fisher exact test. The 95% CIs were calculated using the standard formula for CI for ORs. Due to small sample size in some cases, the contingency tables contained zero values and therefore produced 0 or infinite ORs. To calculate an estimated OR in these cases, a value of 0.5 was added to each value in the 2×2 contingency tables.

**Table.**
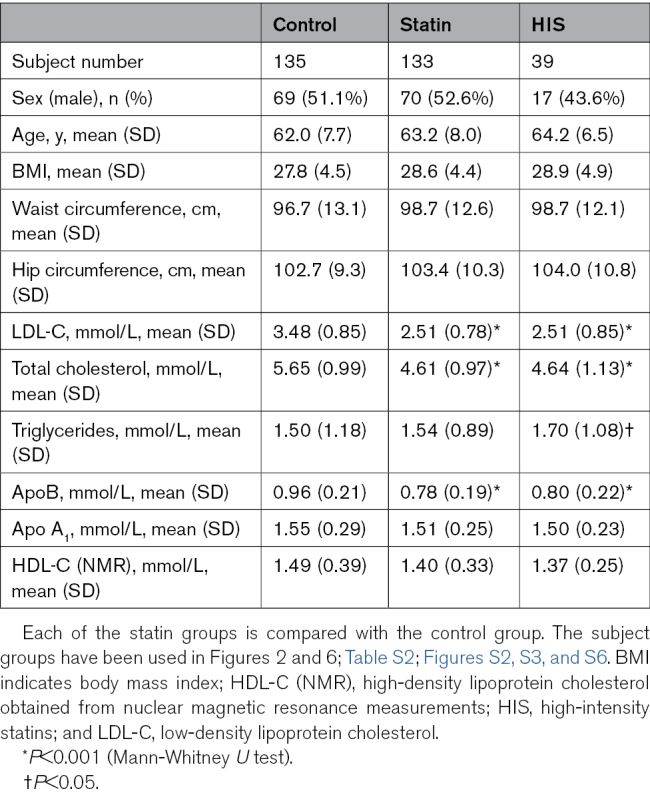
Baseline Characteristics of the Analyzed Subject Groups

**Figure 1.**
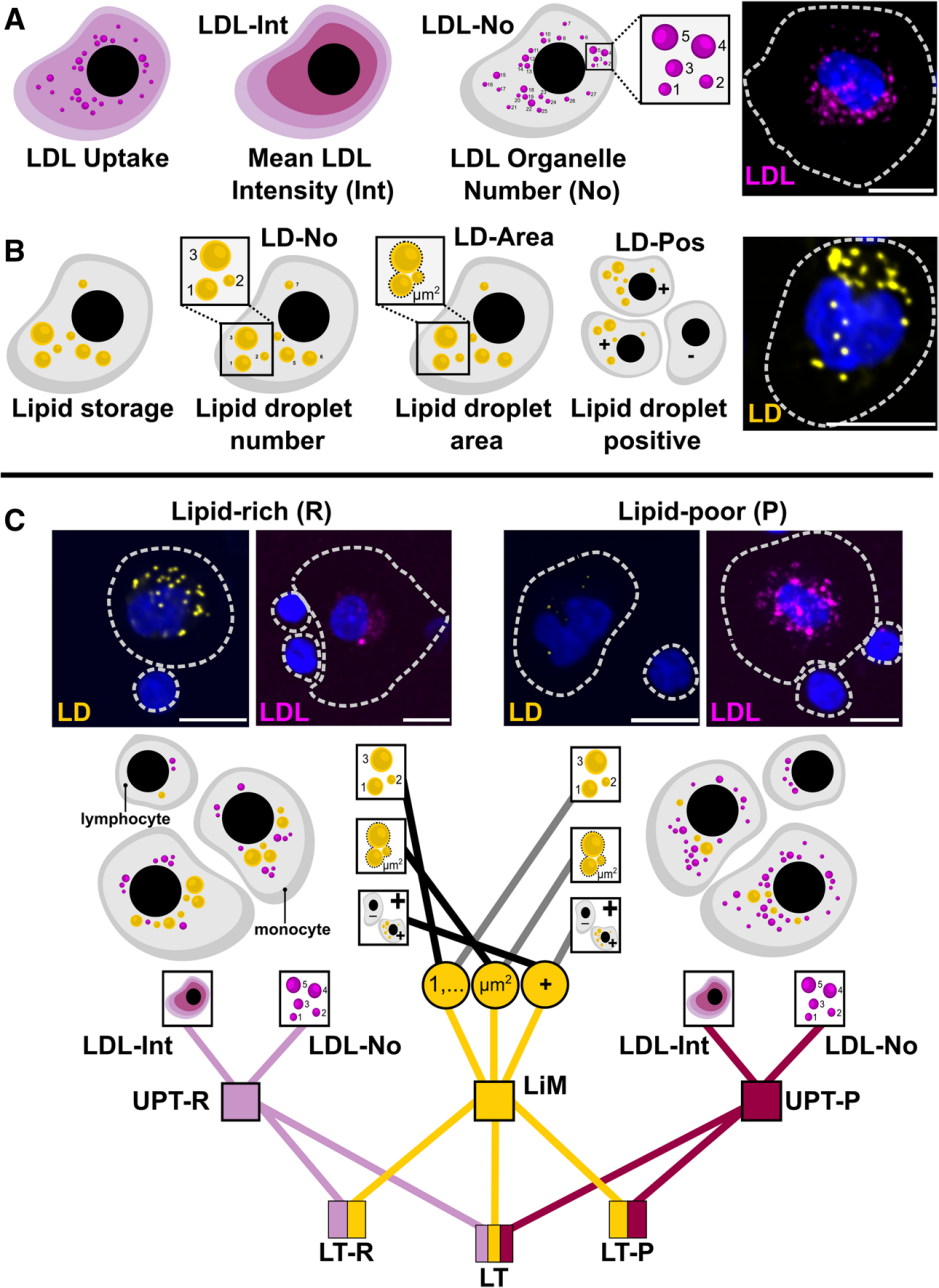
**Multiparametric readouts for cellular lipid storage and trafficking. A**, Schematic presentation of fluorescent LDL (low-density lipoprotein) uptake in cells and quantification as mean cell intensity (LDL-Int) and the number of LDL-filled organelles (LDL-No), together with a representative picture for monocyte LDL uptake in lipid-poor (P) conditions. **B**, Quantification of cellular lipid storage by determining the number of lipid droplets (LD-No), the total lipid droplet area (LD-Area), and the percentage of lipid droplet–positive cells (LD-Pos), together with a representative picture for monocyte lipid droplets in lipid-rich (R) conditions. **C**, Each of these readouts was measured in lipid-rich condition (**left**) and lipid-poor condition (**right**) in monocyte and lymphocyte populations. First, primary uptake readouts for lipid-rich and -poor conditions were combined into uptake scores (uptake score for lipid-rich condition [UPT-R] and uptake score for lipid-poor condition [UPT-P]) and lipid mobilization score (LiM) was calculated by combining the ratios of lipid storage parameters in lipid-rich to -poor conditions. Then uptake and mobilization scores were further combined into lipid trafficking (LT) scores (**C**). Representative pictures in **C** demonstrate lipid droplets and LDL uptake in leukocytes for lipid-rich and -poor conditions. Scale bar=10 µm.

**Figure 2.**
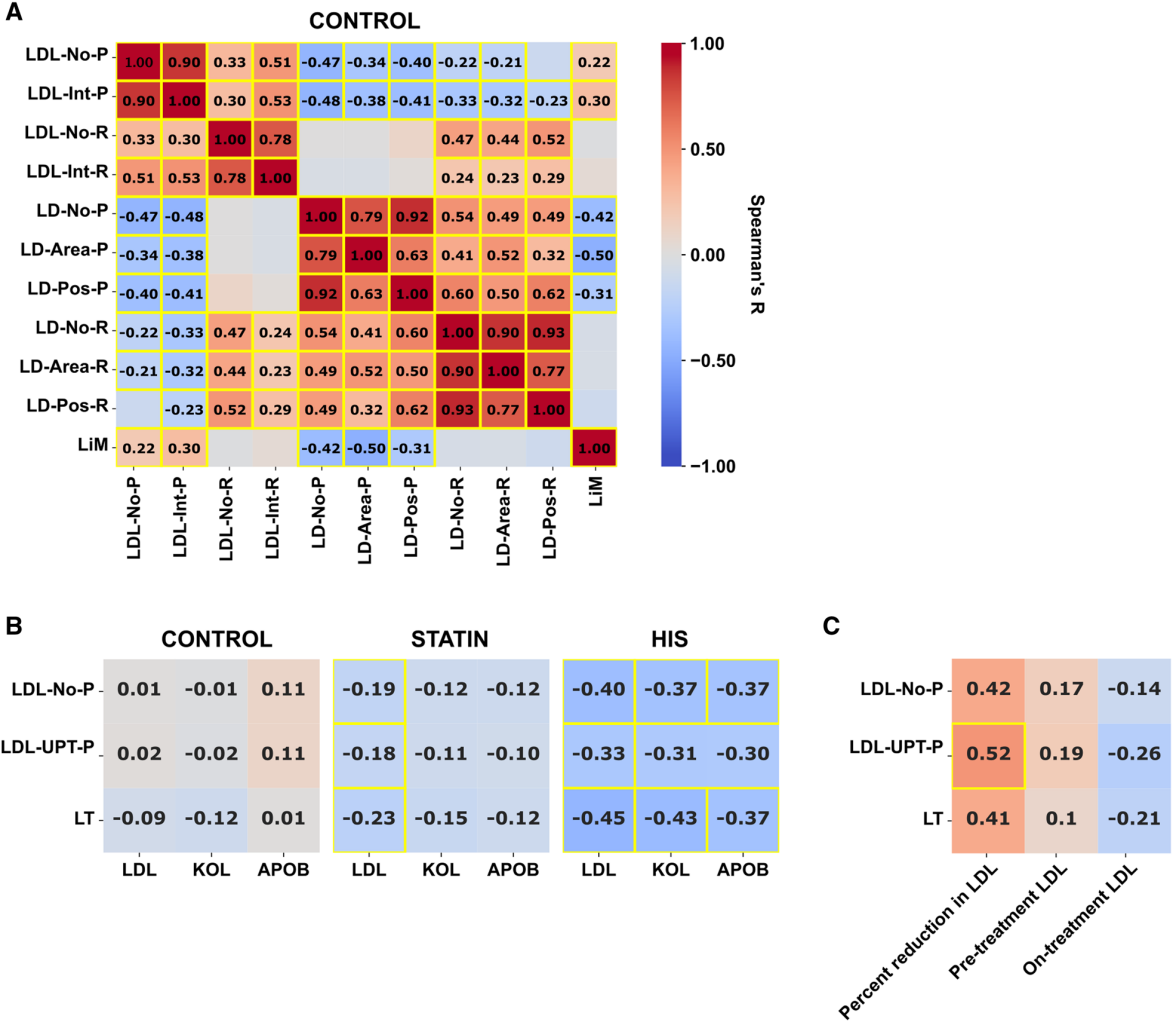
**Cellular scores are associated with blood lipid markers for statin recipients. A**, Correlation heat map for all primary monocyte LDL (low-density lipoprotein) uptake, lipid storage readouts in lipid-rich and lipid-poor conditions together with the lipid mobilization score for subjects of the control group (n=135, yellow boxes indicate *P*<0.05; correlations for statin and high-intensity statins [HIS] groups as well as lymphocytes are contained in Figure S2). **B**, Monocyte readouts such as LDL-filled organelle number in lipid-poor conditions (LDL-No-P) and the combined lipid trafficking score (LT) correlate negatively with circulating low-density lipoprotein cholesterol (LDL-C), total cholesterol (KOL), and APOB in statin and HIS subgroups (controls n=135, statin n=133, HIS n=39). **C**, Correlation heat map for percent reduction in LDL-C, pretreatment, and on-treatment LDL-C for 19 control subjects who started HIS during the follow-up period between 2012 and 2019. (Spearman rank-order correlation coefficients and corresponding asymptotic *P* values). This figure is accompanied by Figures S2, S3, S4; Table. LD-Area-R/P indicates total lipid droplet area; LD-No-R/P, number of lipid droplets; LD-Pos-R/P, percentage of lipid droplet-positive cells; LDL-Int-R/P, mean cell intensity; LDL-No-R, LDL-filled organelle number in lipid-rich conditions; LDL-UPT-R/P, LDL uptake score, all in lipid-rich (R) or lipid-poor (P) conditions; and LiM, lipid mobilization score.

## Results

### Turning Multiparametric Readouts Into Cellular LT Scores

PBMC samples were analyzed with an automated pipeline for detailed quantification of LDL uptake and lipid storage as described previously.^25^ In the LDL uptake assay, we quantified the mean intensity (LDL-Int) of internalized fluorescently labeled LDL and the number of LDL-filled organelles (LDL-No) in lymphocyte-enriched and monocyte-enriched populations (hereafter referred to as lymphocytes and monocytes) under lipid-rich (R) and -poor (P) conditions (Figure 1A and 1C). Although mean cell intensity reflects the total amount of internalized and surface-bound LDL particles, the number of organelles describes exclusively internalized LDL. In lipid-rich conditions, we read out the cellular uptake potential in nonstimulated conditions. When cells are challenged by lipid starvation, LDLR expression is upregulated, increasing LDL uptake from the media. In these conditions, we quantify a maximal LDL uptake capacity of a person’s leukocytes. Mean intensities and organelle counts were normalized to controls and combined for each condition into an uptake score (UPT) for lipid-rich- and -poor conditions by computing the mean of the 2 scores within each condition (Figure 1C).

In the lipid storage assay, we analyzed lipid droplets in lipid-rich and -poor conditions, quantifying the number of lipid droplets (LD-No) and the total lipid droplet area (LD-area) in every lymphocyte and monocyte as well as the percentage of lipid droplet–positive cells (LD-Pos) for lymphocyte and monocyte populations (Figure 1B). Both lymphocytes and monocytes tended to deplete the lipids stored in lipid droplets when challenged with lipid-poor conditions. We expressed this by dividing the values of lipid droplet parameters in lipid-rich and -poor conditions and further computing the mean over these 3 values to form a combined LiM (Figure 1C). Moreover, we combined lipid mobilization and LDL uptake scores into composite LT scores for lipid-rich (LT-R) and -poor (LT-P) conditions by computing the mean over the 2 scores within each condition. The LiM and the LDL uptake scores in both lipid-rich- and -poor conditions were averaged into a single LT score (Figure 1C). This enabled us to look at individual cellular parameters as well as their combinations when integrating the data with physiological and clinical outcomes.

The reliability of the LDL uptake and LD storage parameters were assessed in a previous study,^25^ where the intraindividual variation of these readouts was shown to be low.

### Subject Group Characteristics

We started the analysis by selecting 200 recipients of lipid-lowering therapy and 200 matched controls from the FINRISK 2012 Study for whom PBMC samples were stored in the Biobank. Relevant cellular readouts could be obtained for 135 control subjects and 133 recipients of statin monotherapy (Figure S1; Table). Previously, we observed that leukocyte LDL uptake parameters correlated with achieved LDL-C only for patients with F receiving statin medication.^25^ Statins block cholesterol synthesis, resulting in cellular cholesterol depletion, and elevated LDL catabolism through cellular LDL uptake. Therefore, we expected that different statin potencies might influence our results when we link cellular readouts with circulating lipid values. Many subjects in our cohort were taking low or moderate-intensity statin as per classical definition. To form a statin intensity subgroup enriched with subjects receiving HIS medication we relied on previously reported real-world LDL-C reductions for different doses of simvastatin, atorvastatin, and rosuvastatin.^35–37^ We included recipients of statins with the potential to lower LDL-C by >40% in the HIS group, representing 39 subjects (Figure S1).

The percentage of males in the formed groups was 44% to 53% (Table). There was no significant difference in BMI or waist/hip circumference between the statin groups and controls (Table). As expected, subjects receiving statins had lower total cholesterol (4.61 mmol/L, statin versus 5.65, controls), LDL-C levels (2.51 mmol/L, statin versus 3.48, controls), and ApoB (0.78 mmol/L versus 0.96 mmol/L, controls; Table). In addition, subjects on high-intensity statin monotherapy had higher triglyceride levels than the controls (1.70 mmol/L versus 1.50 mmol/L).

### Relationship of Cellular LDL Uptake and Lipid Droplet Readouts for Control, Statin, and HIS Subject Groups

First, we analyzed the correlations between individual monocyte parameters for LDL uptake, lipid storage, and lipid mobilization in control subjects (Figure 2A). As expected, individual LDL uptake readouts derived from the same treatment condition were highly correlated, which was similar to lipid droplet readouts. On the contrary, correlating LDL uptake or lipid droplet readouts from lipid-rich conditions with the same readouts from lipid-poor conditions provided weaker associations (eg, LDL uptake: LDL-filled organelle number in lipid-poor conditions [LDL-No-P] with LDL-filled organelle number in lipid-rich conditions [LDL-No-R], *R*=0.33; *P*=0.001; lipid droplets: LD-No-P with LD-No-R, *R*=0.54; *P*<0.001), indicating individual differences for LDL uptake and lipid storage readouts in response to the different treatment conditions.

Different patterns were observed for correlations of lipid droplets with LDL uptake readouts in lipid-rich and -poor conditions. In lipid-poor conditions, low lipid droplet abundance correlated with higher LDL uptake (LDL-No-P with LD-No-P: *R*=−0.47; *P*<0.001) and with a higher LiM. On the other hand, lipid droplet readouts from lipid-rich conditions did not correlate with the LiM, showed only a weak correlation with LDL uptake readouts in lipid-poor conditions, and displayed a positive correlation with LDL uptake in lipid-rich conditions. Similar correlation patterns could be observed for lymphocytes, and the statin and HIS groups (Figure S2). However, lymphocyte correlations were overall weaker, and the correlations of lipid droplets with LDL uptake readouts in R conditions were not maintained.

### Association of LT Scores With LDL-C, ApoB, and Total Cholesterol

Next, we investigated how the different leukocyte readouts associated with circulating lipid markers such as LDL-C, total cholesterol, APOB triglycerides, and apoA for the control, statin, and HIS groups (Figure 2B; Figure S3). Similar to our previous observations from patients with FH, monocyte LDL uptake parameters showed a negative correlation with plasma LDL-C for statin recipients (Figure 2B; Figure S3).^25^ This correlation was more pronounced for the quantification of LDL-filled organelles (LDL-No; Figure 2B) as compared with cellular LDL intensity (mean intensity of internalized fluorescently labeled LDL; Figure S3) when measured in P condition. Stronger associations were also seen for measurements derived from P conditions as compared with R conditions (Figure 2B; Figure S3) and when quantified in monocytes as compared with lymphocytes (Figure S3). The correlation strength for LDL-No, LDL uptake score (LDL-UPT), and LT with LDL-C increased in the HIS group. Similar correlations were obtained for total cholesterol and APOB (Figure 2B). For the HIS group, LDL-No-P explained 16% of the variability of the circulating LDL-C (coefficient of determination *R*^2^=0.16; *P*=0.01), 14% of the total cholesterol (*R*^2^=0.14; *P*=0.02), and 13% of APOB (*R*^2^=0.13; *P*=0.02).

Combination of the lipid uptake scores with the LiM into the LT score (Figure 1C) provided stronger associations with achieved LDL-C than LDL-No-P, with a further strengthening of the correlation in the HIS group (Figure 2B), explaining 21% of the variability of LDL-C for the HIS group (*R*^2^=0.21; *P*=0.004).

Although pretreatment lipid values were not available for the statin and HIS groups, we managed to identify 22 control subjects who started lipid-lowering therapy and received atorvastatin or rosuvastatin between 2012 and 2019. Of these subjects, 6 individuals first received simvastatin treatment and were moved to atorvastatin/rosuvastatin later on. Sixteen individuals were directly moved from naive to atorvastatin/rosuvastatin. Importantly, although in some cases the medication was received several years after the leukocyte isolation, there was a statistically significant correlation between LDL-UPT and percent reduction in LDL-C (*R*=0.52, *P*=0.022; Figure 2C). Also, LDL-No and LT scores tended to be correlated with the percent reduction in LDL-C (*R*=0.42, *P*=0.073; *R*=0.41, *P*=0.149; Figure 2C). The correlation with achieved LDL-C was weaker. When looking at all the individual cellular readouts and their correlation with the percent reduction, achieved LDL-C, and pretreatment LDL-C (Figure S4), we also noticed a significant correlation between the mean intensity of internalized fluorescently labeled LDL and the percent reduction in LDL-C (Figure S4).

### LT Scores Associate With a Proatherogenic Lipoprotein Profile

To investigate whether alterations of cellular LT impacts also other lipid particles and species, we extended our study with NMR metabolomics, providing access to 213 biomarkers, including the concentration and composition of 14 lipoprotein particle classes.

**Figure 3.**
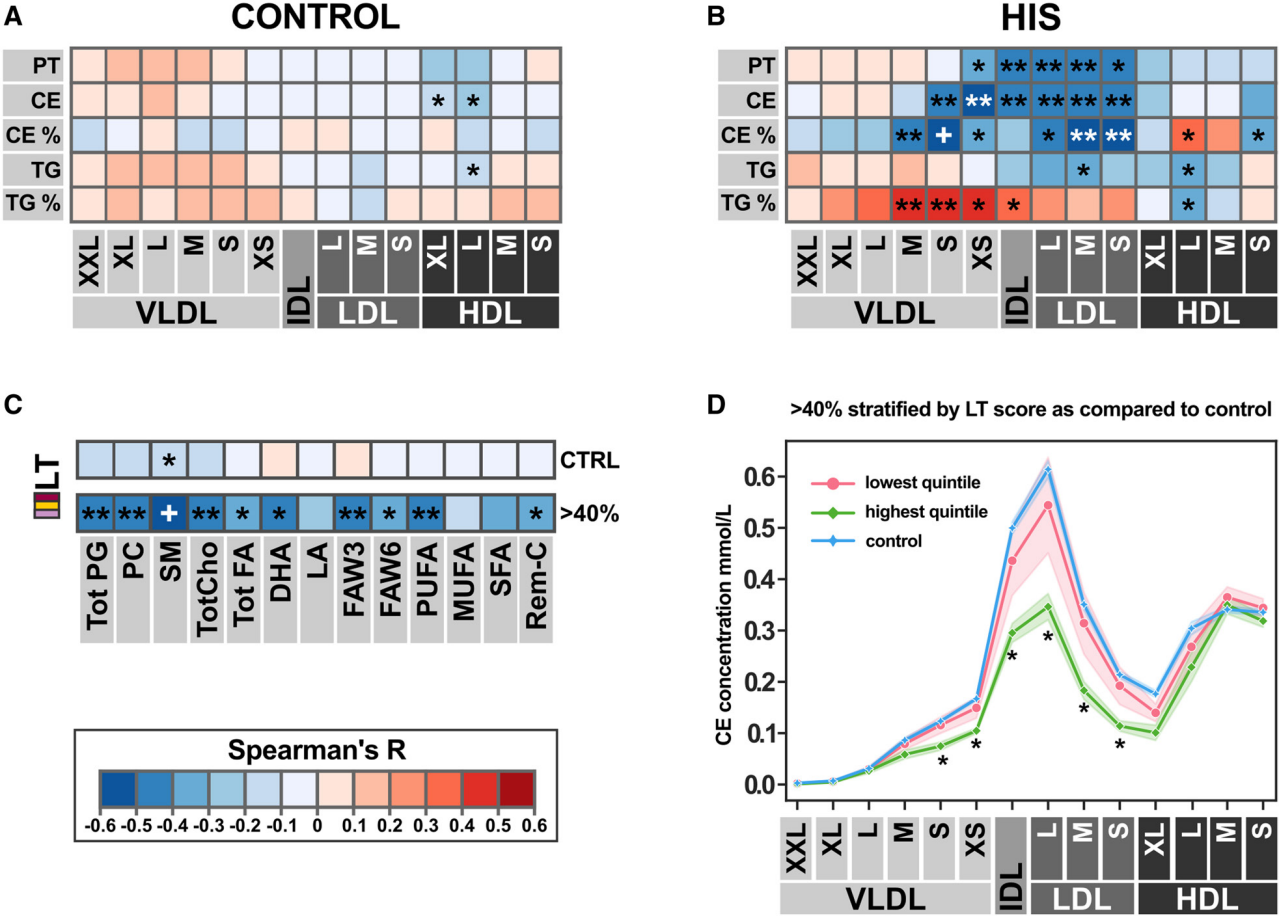
**Low lipid trafficking (LT) scores are associated with a proatherogenic lipoprotein profile for high-intensity statin (HIS) recipients. A**, Correlation heatmap of combined LT score to different lipid concentrations in 14 lipoprotein subclasses in control subjects. Heat map colors correspond to *r*_*s*_ range and are explained at the **bottom left** corner of the figure. **B**, Correlation heat map of LT score to 14 lipoprotein subclasses in the HIS recipients group. **C**, Correlation heatmap of LT score to circulating concentration of different lipid species in control individuals vs HIS group. The species are as follows: total phosphoglycerides (Tot PG), phosphatidylcholine and other cholines (PC), sphingomyelins (SM), total cholines (TotCho), total fatty acids (Tot FA), 22:06 docosahexaenoic acid (DHA), 18:02 linoleic acid (LA), omega-3 and omega-6 fatty acids (FAW3 and FAW6), polyunsaturated fatty acids (PUFA), monounsaturated fatty acids (MUFA), saturated fatty acids (SFA), and remnant cholesterol (Rem-C). **D**, The lowest quintile of LT scores for high-intensity statin users shows a cholesterol ester profile which is similar to controls, while subjects within the highest quintile have significantly reduced CE concentrations. *P<0.05, **P<0.01, +*P*<0.001 (**A** through **C**) Spearman rank-order correlation coefficients and corresponding asymptotic *P* values, (**D**) Mann-Whitney *U* test). Subject group sizes: control n=135, statin n=131, HIS n=38. This figure is accompanied by Table S2. CE% indicates percent of cholesterol ester in particles; CE, cholesterol ester concentration; CTRL, control; HDL, high-density lipoprotein; IDL, intermediate-density lipoprotein; L, large particles; LDL, low-density lipoprotein; M, medium particles; PT, total particle concentration; S, small particles; TG%, percent of triglycerides in particles; TG, triglyceride concentration; VLDL, very low-density lipoprotein; XS, extra-small particles; and XXL, extra-extra-large particles.

Based on our observations with LDL-C we utilized the LT score for correlations with the NMR metabolomics data. For control subjects, LT score showed a weak negative association with cholesterol ester and triglyceride concentration in large HDL (high-density lipoprotein) particle fractions (Figure 3A) and with sphingomyelin (Figure 3C). For the HIS group, the LT score correlated negatively with XS-VLDL, IDL (intermediate-density lipoprotein), L-LDL, M-LDL, and S-LDL particle concentrations (Figure 3B). Higher cholesterol ester concentrations, in particles from S-VLDL to S-LDL were observed for subjects from the HIS group with a low LT score, whereas associations with triglycerides were less pronounced (Figure 3B). We also observed correlations with the % lipid distribution across lipid particles. HIS subjects with lower LT score displayed a higher share of cholesterol ester in small VLDL (very low-density lipoprotein), IDL, and LDL particles, whereas in large and medium-sized HDL particles the share dropped (Figure 3B). Vice-versa, % triglycerides were lower in small VLDL and IDL particles for subjects with low LT scores from the HIS group (Figure 3B).

Besides cholesterol esters, LT score also negatively correlated with sphingomyelin concentration for the HIS group (Figure 3C). Other lipid species were also increased for subjects with low LT, but association was less profound as compared with sphingomyelin.

Interestingly, HIS subjects within the highest quintile of the LT score had substantially lower cholesterol ester concentrations in S-VLDL, XS-VLDL, IDL, and LDL particles (Figure 3D), as compared with those within the lowest quintile. Moreover, cholesterol ester concentrations across the different lipoprotein classes were similar for HIS subjects within the lowest LT quintile and subjects without cholesterol-lowering medication (Figure 3D).

### LT Scores Integrated With Polygenic Risk Scores

**Figure 4.**
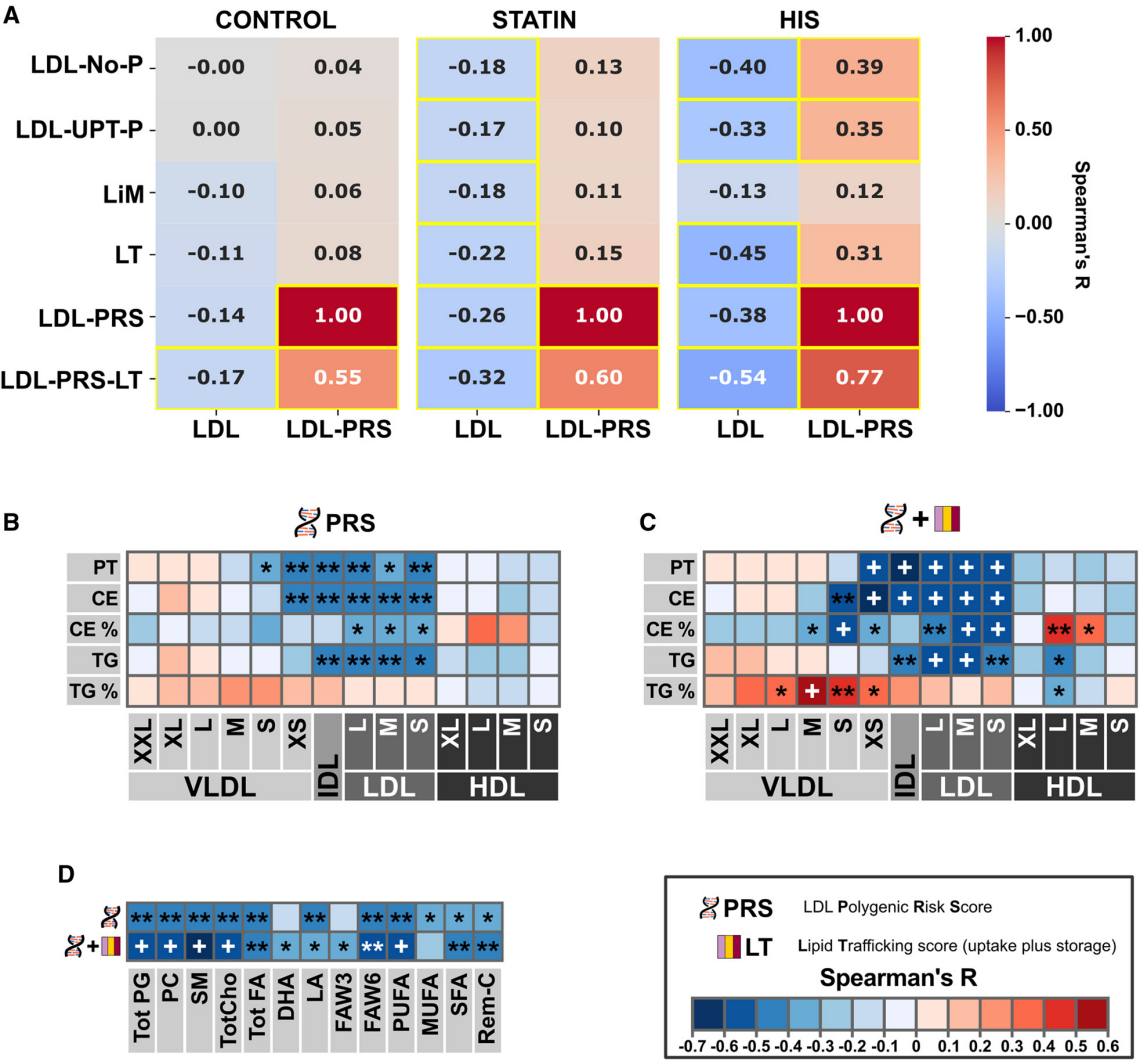
**Cellular scores can be combined with low-density lipoprotein cholesterol polygenic risk score (LDL-PRS) to improve the associations with low-density lipoprotein cholesterol (LDL-C) and other lipids. A**, Correlation heat map displaying the relationship of monocyte LDL uptake, lipid mobilization, lipid trafficking (LT), LDL-PRS, and a combined score of LT and LDL-PRS (LDL-PRS-LT) with achieved LDL-C and the LDL-PRS for control, statin, and high-intensity statins (HIS) groups. Control n=134, statin n=131, HIS n=39, correlations with *P*≤0.05 are encircled in yellow. **B**, Correlation heatmap of LDL-PRS score to 14 lipoprotein subclasses in high-intensity statin (HIS) group. Heatmap colors correspond to Spearman rank range and are explained at the **bottom right** corner of the figure. **C**, Correlation heatmap of combined score of LDL-PRS with LT to 14 lipoprotein subclasses in HIS recipients. **D**, Correlation heatmap of LDL-PRS score and combined LDL-PRS-LT score to circulating concentration of different lipid species in the HIS group. The species are as follows: total phosphoglycerides (Tot PG), phosphatidylcholine and other cholines (PC), sphingomyelins (SM), total cholines (TotCho), total fatty acids (Tot FA), 22:06 docosahexaenoic acid (DHA), 18:02 linoleic acid (LA), omega-3 and omega-6 fatty acids (FAW3 and FAW6), polyunsaturated fatty acids (PUFA), monounsaturated fatty acids (MUFA), saturated fatty acids (SFA), and remnant cholesterol (Rem-C). *P<0.05, **P<0.01, +*P*<0.001 (Spearman rank-order correlation coefficients and corresponding asymptotic *P* values). Subject group sizes in **B** through **D**: control n=134, statin n=129, HIS n=38. This figure is accompanied by Table S3. CE% indicates percent of cholesterol ester in particles; CE, cholesterol ester concentration; CTRL, control; HDL, high-density lipoprotein; IDL, intermediate-density lipoprotein; L, large particles; LDL, low-density lipoprotein; LDL-No-P, LDL-filled organelle number in lipid-poor conditions; LDL-UPT-P, LDL uptake score in lipid poor conditions; LiM, lipid mobilization score; M, medium particles; PT, total particle concentration; S, small particles; TG%, percent of triglycerides in particles; TG, triglyceride concentration; VLDL, very low-density lipoprotein; XS, extra-small particles; and XXL, extra-extra-large particles.

To investigate the relationship of leukocyte lipid metabolism readouts with the polygenic risk score for LDL (LDL-PRS), we computed correlations between selected cellular readouts (LDL-No-P, LDL-UPT-P, LiM, and LT) and LDL-PRS and LDL-C within each subject group (control, statin, and HIS; Figure 4A). We also computed the correlation between the LDL-PRS and LDL-C. The uptake scores LDL-No-P and LDL-UPT-P, LiM, and LT did not correlate with the LDL-PRS in the control group (Figure 4A), which is similar to our previous results.^25^ Importantly, the correlation strengths increased in the statin and especially in the HIS group with the number of LDL-filled organelles (LDL-No-P) and the combined LDL uptake score (LDL-UPT-P) in lipid-poor conditions correlating with LDL-PRS (*R*=0.39, *P*=0.012; *R*=0.35, *P*=0.027; Figure 4A). The LDL-PRS correlated with LDL-C in statin users and the association strength further increased in HIS subjects (Figure 4A). Based on these results, we evaluated whether the combination of the LT score with the LDL-PRS would lead to a better association with LDL-C as compared with the individual scores. The combined score (combined score of LT and LDL-PRS) showed significant associations with LDL-C for control subjects (*R*=−0.17, *P*=0.050) and increased in association strength in the statin (*R*=−0.32, *P*<0.001) and HIS groups (*R*=−0.54, *P*<0.001; Figure 4A). For the HIS group, the combined score explained 29% of the LDL-C variability (*R*^2^=0.292; *P*<0.001), a 41% increase over the LT score (*R*^2^=0.207, *P*=0.004; Figures 2B and 4A) and 99% over the LDL-PRS (R^2^=0.147, *P*=0.016). Then, we extended this analysis to the NMR metabolomic data (Figure 4B through 4D). Similar to the LT score, a poor LDL-PRS associated with proatherogenic lipoprotein particles, with higher cholesterol ester and triglyceride content for subjects of the HIS group (Figure 4B). Although the % distribution of cholesterol esters and triglycerides was more affected by the LT score (Figure 4B; Figure 3B) combination of the LDL-PRS with the LT score further improved the association strength with the proatherogenic lipoprotein profile (Figure 4C), explaining, for example, up to 37.7% of the variability (*R*^2^=0.377; *P*<0.001) of cholesterol ester concentration in XS-VLDL particles of HIS subjects.

### LT Scores Enable Stratification for LDL Target Level Attainment

**Figure 5.**
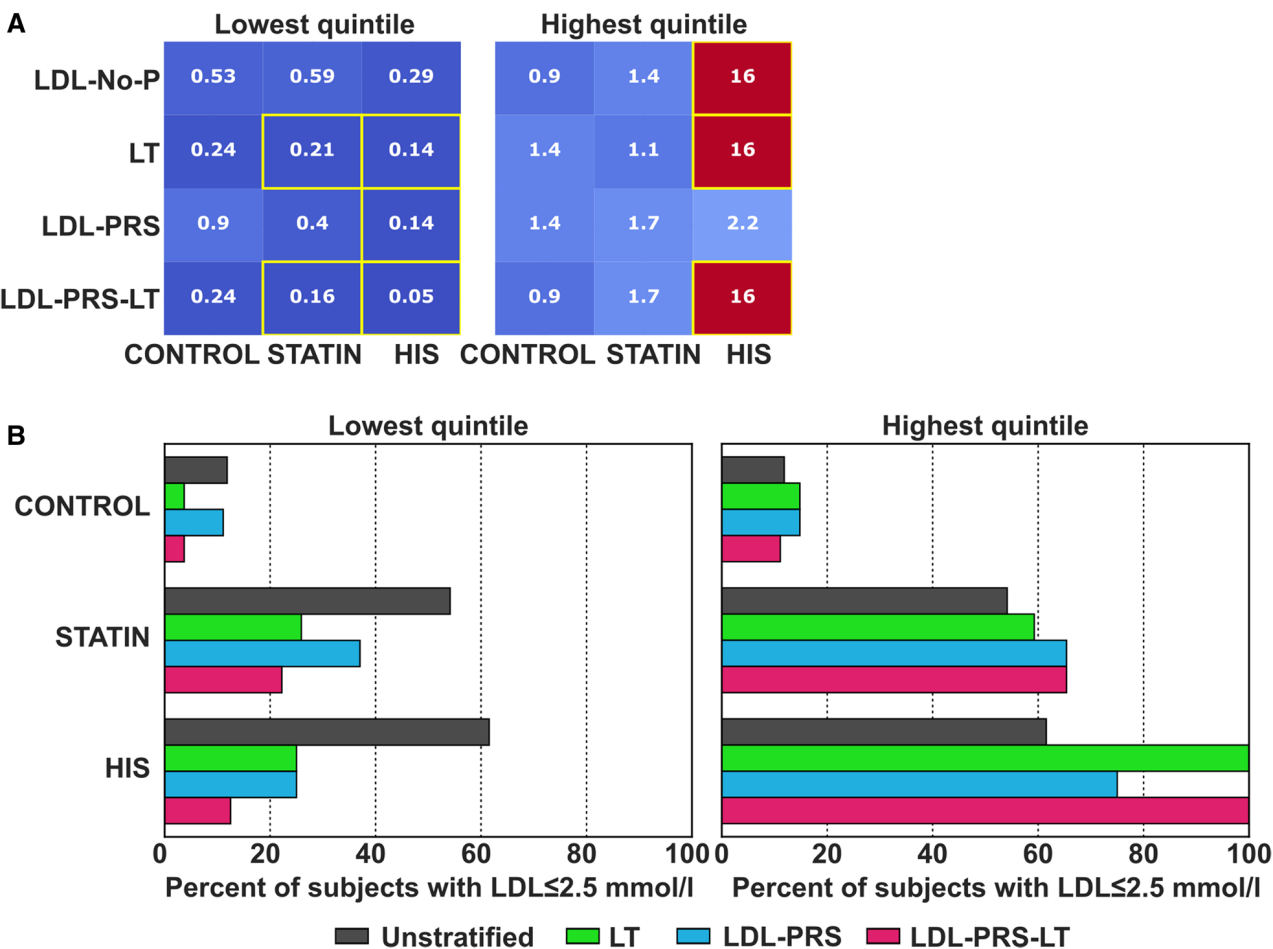
**To stratify subjects for low-density lipoprotein cholesterol polygenic risk score (LDL-PRS) goal attainment. A**, Heat map displaying the odds ratios for the association of LDL-filled organelle number in lipid-poor conditions (LDL-No-P), lipid trafficking (LT), LDL polygenic risk score (LDL-PRS), and LDL-PRS+LT with achieving an LDL cholesterol (LDL-C) target of 2.5 mmol/L for the lowest and the highest quintiles of the scores compared with the rest of the group. Odds ratios have been calculated separately for the control, statin, and high-intensity statins (HIS) groups. Statistically significant odds ratios are highlighted in yellow (*P*<0.05, Fisher exact test). The CIs and *P* values are listed in Table S4. **B**, **left**, Percent of subjects achieving an LDL-C goal of 2.5 mmol/L when being within the lowest quintile of the LT score (green), LDL-PRS (blue), or combined LDL-PRS-LT score (magenta) as compared with subjects without stratification (gray) for control, statin, and HIS groups. **Right**, Percent of subjects achieving an LDL-C target levels of 2.5 mmol/L for subjects within the highest quintile of LT (green), LDL-PRS (blue), and combined LDL-PRS-LT as compared with subjects without stratification for control, statin, and HIS groups. The control group had 134 subjects (27 in focus quintiles), the statin group had 131 subjects (27 in focus quintiles), and the HIS group consisted of 39 subjects (8 in focus quintiles). This figure is accompanied by Figure S5; Table S4.

Next, we investigated whether LDL uptake, lipid mobilization, and LT scores as well as LDL-PRS could enable the definition of subject subgroups who are at higher or lower odds to achieve a predefined LDL-C treatment goal (Figure 5, Figure S5). Estimated OR were used for those subgroups in which all subjects achieved or did not achieve the target goals (Figure 5A, Figure S5). The 2011 ESC/EAS guidelines^53^ were in use during the sample collection: The target goals for LDL-C were <2.5 mmol/L for high cardiovascular risk and <1.8 mmol/L for very high cardiovascular risk. Individuals in each subject group were divided into quintiles for the different cellular scores and we evaluated whether individuals within the highest or lowest quintile achieved an LDL-C target level of 2.5 mmol/L. In general, subjects within the lowest quintile for LDL uptake, lipid mobilization, or LT scores were less likely to achieve the <2.5 mmol/L target goal, which was more pronounced for the HIS group than for the statin group (Figure 5, Figure S5). However, the LT scores LT (OR, 0.24, *P*=0.193 for controls; OR, 0.21, *P*<0.001 for statin; and OR, 0.14, *P*=0.037 for HIS), LT-R, and LT-P provided more consistent results than the individual LDL uptake readouts (Figure 5; Figure S5). Of the subjects within the lowest quintile of the LT score, 3.7% in the control group, 25.9% of the statin, and 25.0% of the HIS group achieved LDL-C <2.5 mmol/L (Figure 5B). Therefore, goal attainment for subjects within the lowest quintile of the LT score is >2-fold lower than for unstratified individuals for which 11.9% achieved LDL-C <2.5 mmol/L for the control group, 54.9% for the statin, and 61.5% for the HIS group. LDL-PRS alone did not perform as good as the LT scores for stratifying subjects on whether they achieved a goal of <2.5 mmol/L LDL-C (Figure 5A and 5B). However, combination of the LT score with the LDL-PRS further improved the discrimination on whether subjects within the lowest quintile did achieve LDL-C <2.5 mmol/L (combined score of LT and LDL-PRS: 3.7%, OR, 0.24, *P*=0.193 control; 22.2%, OR, 0.16, *P*<0.001 statin; 12.5% OR, 0.05, *P*=0.003 HIS; Figure 5A and 5B; Figure S5).

Those subjects within the highest quintile of LDL uptake or LT scores were more likely to achieve the LDL-C <2.5 mmol/L goal (Figure 5, Figure S5). For the HIS group, all subjects within the highest quintile of the LT score achieved LDL-C <2.5 mmol/L (LT, 100%; OR_est, 16; *P*=0.015), whereas only 75% achieved the goal for the highest quintile of the LDL-PRS (LDL-PRS, 75%; OR, 2.2; *P*=0.450). In this case, combining LT scores with LDL-PRS did not result in further improvements, except for combining LDL-PRS with LT-R (OR, 5.21, *P*=0.004 for controls; OR, 6.1, *P*=0.001 for statin; and OR_est, 16, *P*=0.015 for HIS; Figure S5).

The CIs and *P* values of ORs for achieving an LDL-C level of 2.5 mmol/L are shown in Table S4. For more stringent target goals of <1.8 and 1.4 mmol/L, our cellular LDL uptake and LT scores provided similar tendencies as for the 2.5 mmol/L goal. However, as only few individuals achieved these targets the OR calculations were less reliable (data not shown).

### LT Scores and CVD

During a 7-year follow-up of the 265 subjects, 11 (4 in controls and 7 in the statin group) were diagnosed with myocardial infarction and 10 with stroke (5 in each of the 2 groups), resulting in 21 cardiovascular events. This allowed us to get a first impression of whether cellular scores could aid in cardiovascular risk assessment.

**Figure 6.**
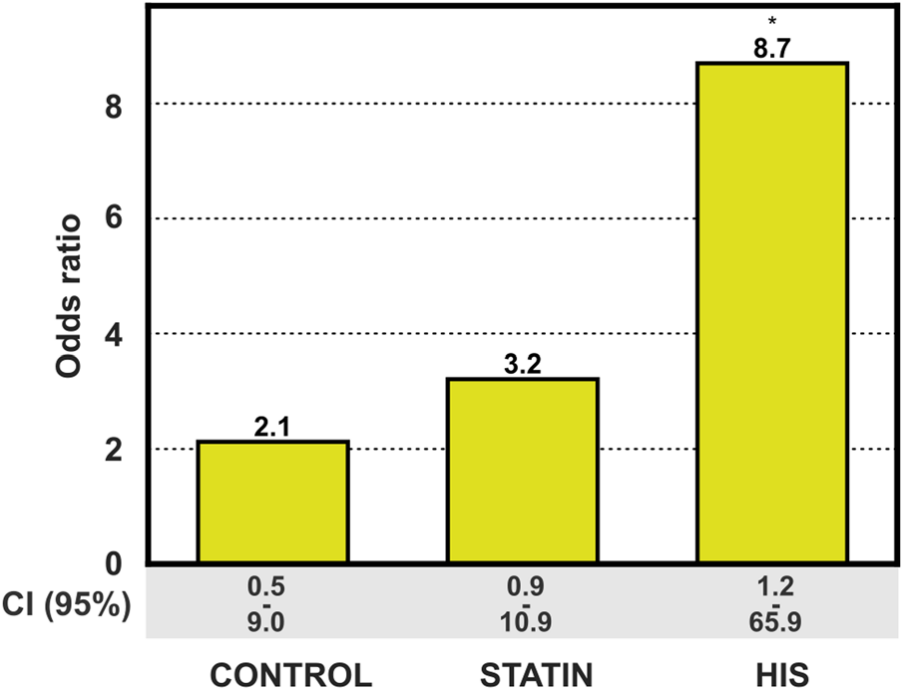
**Lipid mobilization score in risk assessment for myocardial infarction and stroke.** Subjects in the lowest quintile of lipid mobilization score have increased odds of experiencing a cardiovascular event as compared with the rest of the group. The odds ratio is increasing in the statin and high-intensity statin (HIS) group. The control group had 135 subjects (27 in focus quintile), the statin group had 133 subjects (27 in focus quintile), and the HIS group consisted of 39 subjects (8 in focus quintile). *P<0.05 (Fisher exact test). This figure is accompanied by Figure S6.

Interestingly, the likelihood of experiencing a CVD event during the follow-up increased in control, statin, and HIS groups for subjects with a LiM within the lowest quintile (control OR, 2.1, *P*=0.383; statin OR, 3.2, *P*=0.067; and HIS OR, 8.7, *P*=0.049; Figure 6). These effects were also visible for individual LiMs (LD-No–fold, LD-area–fold, and percentage of lipid droplet–positive cells–fold; Figure S6). Furthermore, HIS subjects within the highest quintile for the LiM did not experience a CVD event, although these effects were not significant (OR_est, 0.28, *P*=0.563; Figure S6). The LiM reflects changes in lipid droplet abundance in lipid-rich and -poor conditions. We observed a tendency that HIS subjects within the highest quintile for the lipid storage readouts (LD-No, total lipid droplet area, and percentage of lipid droplet–positive cells) were more likely to experience a CVD event during the follow-up (LD-Pos-P: OR, 8.7, *P*=0.049; Figure S6). In contrast, individual LDL uptake readouts did not associate with CVD events during the follow-up and only the LT score from lipid-poor conditions (LT-P) resulted in an OR of 30 ([95% CI, 2.65–339.75]; *P*=0.004) for experiencing a CVD event for subjects in the lowest quintile of the HIS group as compared with the rest of the group.

## Discussion

We performed a systematic functional cellular analysis for a set of subjects from a representative, cross-sectional population survey, observing considerable interindividual variability and widespread defects in cellular LDL uptake and lipid mobilization in leukocyte subpopulations. We further combined individual LDL uptake and lipid mobilization readouts into one LT score. For subjects receiving HIS medication, lower cellular LDL uptake and LT score were associated with higher circulating concentrations of LDL-C, IDL, small VLDL particles, and cholesterol ester content across APOB-containing lipid particle subclasses. Elevated LDL-C leads to the formation of atherosclerotic plaques and the progression of CVD.^1^ Besides LDL, small VLDL and IDL can cross the endothelial cell barrier and remnant cholesterol is a known driver of atherosclerosis.^54,55^ We further observed an inverse association between the LT score and sphingomyelin concentration in HIS recipients. A higher amount of sphingomyelin in LDL particles renders them more prone to aggregation and stimulates atherosclerosis.^56^ It appears that subjects with poor LT scores have limited capacity to benefit from statin monotherapy, which is also reflected in them being at higher odds for experiencing an adverse cardiovascular event when compared with others on equivalent medication. Statins act by blocking cholesterol synthesis and elevating the expression of liver LDL receptors.^16,32^ Subjects with reduced cellular capacity to uptake LDL and to utilize stored lipids may have a limited ability to upregulate LDLR and consequently efficiently clear proatherogenic lipoproteins upon statin therapy. This reasoning can be also supported by our observation that the association of LDL uptake and LT score with LDL-C is strengthening with higher statin intensity, reflecting greater reliance of subjects on LDL catabolism in such conditions.

The role of lipid accumulation in circulating monocytes in the development of Atherosclerotic CVD is receiving increased attention. Patients with FH display increased lipid accumulation in circulating monocytes.^21,22^ In mouse models, lipid-laden circulating monocytes showed upregulation in CD11c and an increased potential to invade nascent atherosclerotic lesions.^23^ Several studies in humans align with these findings, for example, a postprandial increase in monocyte lipid droplets associated with proinflammatory features of the same cells.^57^

In our cohort, we saw high interindividual variability in regard to monocyte lipid droplet content. Moreover, we observed large differences in the rate with which lipid droplets declined when monocytes were challenged with a lipoprotein-depleted medium (lipid mobilization). Strikingly, individuals with low lipid droplet depletion in lipoprotein-depleted medium, and a resulting low LiM had several times higher OR for myocardial infarction or stroke as compared with the rest of their group and the effect was more pronounced with an increase in statin intensity. Our data indicate that combining LiM score with LDL uptake readouts may improve residual risk stratification, especially for people who are already receiving HIS treatment, however, a low incidence of CVD in our cohort is an important limiting factor and a specific study addressing this question would be needed. Nevertheless, our findings are well in line with previous reports, suggesting that monocyte lipid accumulation in the circulation is associated with atherosclerotic plaque progression.

Similar to the LT score, association strength of LDL-PRS with LDL-C increased with higher statin intensity. A potential explanation for this could be that a portion of the single nucleotide polymorphisms contained in the LDL-PRS is linked to cellular LDL uptake. Our observation that LDL uptake correlates with the LDL-PRS for HIS recipients supports this hypothesis. Apparently, LDL-PRS and LT score have nonoverlapping features as well, which would explain why a combination of the LDL-PRS with the LT score provided a better association with proatherogenic lipoprotein particles and in some instances improved subject stratification for LDL-C goal attainment. At this stage, it is not clear how most gene loci contained in the LDL-PRS result in elevated LDL-C. Gene loci may impact food intake, lifestyle, intestinal cholesterol uptake but also cholesterol synthesis, and lipoprotein production. A better understanding of the functional mechanisms of how LDL-PRS gene loci contribute to hypercholesterolemia might provide deeper insight into the nonoverlapping features of LDLR-PRS and cellular LDL uptake scores and thereby unravel novel factors contributing to disease progression.

Our observations are supported by genetic studies that link loss-of-function variants of the LDL receptor to a higher incidence of CVD.^58,59^ Although the detection of an LDLR loss-of-function variant implies defective LDL uptake, we directly quantify this cellular process with high-content microscopy of leukocyte subpopulations.

It is known that individuals from the general population can have a cellular LDL uptake similar to heterozygous FH patients with with loss-of-function LDLR variants.^31^ Our study supports these findings and highlights for the first time the physiological and clinical consequences for subjects with LT defects in the general population.

Interindividual variability in treatment outcomes for statin monotherapy was observed before,^3,13,14^ also when statin adherence was monitored closely.^15^ Consequently, it seems unlikely that the interindividual variability only stems from nonadherence. Several studies highlight difficulties in LDL-C goal attainment for patients on statin medication and in making the switch to combination therapy.^2,5^ Systematic quantification of cellular LT pathways could be applied as a precision-medicine assay for treatment predictions. In this case, the cellular test is performed before lipid-lowering therapy is initiated. The test would identify high-risk individuals and patients who should start combination therapy, for example, statin+ezetimibe, immediately, because they do not have sufficient LDL catabolism. This would enable rapid and efficient LDL-C lowering in a short time targeted to subjects with high cardiovascular risk. Our data which links pretreatment LDL uptake readouts with the percent reduction in LDL-C for HIS recipients (Figure 2C) supports such an approach. We envisage that with additional clinical data, it will be possible to extend the treatment recommendation to PCSK9is or combination therapies with other cholesterol and lipid-lowering therapies.

Alternatively, a cellular test could be scheduled after statin treatment has been initiated. This enables the physician to quickly identify individuals who do not achieve their target levels due to a reduced LDL catabolism capacity. Moreover, the physician gains deeper insight into whether a patient is at increased residual CVD risk. This enables immediate prescription of combination therapy for those patients with the highest need. For subjects with high LDL catabolism and no indication of increased residual risk, the cellular test provides validation for statin monotherapy as an efficient treatment strategy. This might be valuable information for patients with statin side effects, encouraging the use of bempedoic acid or trying different statin types to overcome the side effects. An additional advantage of the cellular test is that it may discriminate poor adherence from a poor response and for patients with a good LDL uptake capacity, it can be expected that statin medication will result in substantial LDL-C reductions.

We envisage that cellular readouts can be implemented in a clinical setting similar to other specialized clinical assay tests. In this case, blood samples are collected and shipped to a centralized facility for processing and deriving the cellular readouts. Then the results are returned to healthcare professionals via an online system.

It is important to keep in mind, however, that our current observations for HIS recipients are derived from a limited number of subjects from the Finnish population. This might affect the applicability of these results to other populations, especially those with different ethnic backgrounds. The medication status of the subjects in this study was determined by their drug purchase patterns. Although this method gives a more detailed picture compared with the yes/no type of questionnaire initially available, it can only provide an approximation of real adherence.

Here, we acquired a comprehensive set of functional cell data for 268 individuals from the FINRISK 2012 Study. The analysis strategy is based on a semiautomated workflow with fully automated microscopy and image analysis. This reduces researcher bias, increases the reproducibility of the results, and makes it possible to scale the analysis to higher throughput, enabling clinical applications in the future. This study highlights that intraindividual variation of biological processes should receive more attention in the treatment of hypercholesterolemia.

## Article Information

### Acknowledgments

The samples and sample-related data used for the research were obtained from The National Institute for Health and Welfare (THL) Biobank (study number: THLBB2020_7). The authors thank all study participants for their generous participation at THL Biobank and FINRISK 2012 Study and Tamara Alagirova for valuable comments on the manuscript. We thank the FIMM High-Content Imaging and Analysis (FIMM-HCA) unit for access to microscopy infrastructure. S.G. Pfisterer and I. Hlushchenko designed the study. I. Hlushchenko and M.M. Islam performed experiments and analyzed the cellular data. S. Pamilo and I. Hlushchenko performed data analysis. M. Tamlander and S. Ripatti performed the calculations of polygenic risk scores. S.G. Pfisterer, S. Pamilo, and I. Hlushchenko interpreted the results. S.G. Pfisterer and I. Hlushchenko wrote the manuscript and S. Pamilo revised the manuscript. S. Ripatti and M. Tamlander have critically reviewed the manuscript. All authors have approved the manuscript for publication.

### Sources of Funding

Supported by grants from the Academy of Finland 328861 and 325040, Business Finland (Research to Business) 1821/31/2021, Magnus Ehrnrooth, and the Foundation for Cardiovascular Research to S.G. Pfisterer. Collaborative research project funding from the European Union (EU) Horizon RIA (Research and Innovation Actions): 101155885-2, project FH-EARLY to S.G. Pfisterer at MONCYTE Health, Grants from Academy of Finland Center of Excellence in Complex Disease Genetics 312062, Academy of Finland 285380, the Finnish Foundation for Cardiovascular Research and the Sigrid Jusélius Foundation to S. Ripatti. Funding from the Doctoral program in Population Health, University of Helsinki to M. Tamlander.

### Disclosures

A patent application covering the use of the here-suggested stratification methods has been filed (application number: FI2021050861W·2021-12-10), in which the University of Helsinki is the applicant and S.G. Pfisterer is one of the inventors. The application is currently at the stage of individual filing in selected countries. The technology for the cellular analysis covered in the patent application is used in this manuscript and has also been previously published as a proof-of-concept study for familial hypercholesterolemia patients. The patent has been transferred to the university spin-out company MONCYTE Health. S.G. Pfisterer is a founder of MONCYTE Health and I.A.K. Lähdeniemi, S.G. Pfisterer, and S. Pamilo are affiliated with MONCYTE Health in addition to the University of Helsinki. The other authors report no conflicts.

### Supplemental Material

Supplemental Methods

Tables S1–S4

Figures S1–S6

Major Resources Table

STROBE Checklist
